# Editorial: The neural mechanisms involved in mood disorder-sleep disorder interaction

**DOI:** 10.3389/fpsyt.2025.1642372

**Published:** 2025-06-25

**Authors:** Su-Rong Yang, Michael Lazarus, Jun Lu

**Affiliations:** ^1^ School of Basic Medical Sciences, Fudan University, Shanghai, China; ^2^ International Institute for Integrative Sleep Medicine (WPI-IIIS), Tsukuba Institute for Advanced Research (TIAR), and Institute of Medicine, University of Tsukuba, Tsukuba, Japan; ^3^ Stroke Center, The First Hospital, Jilin University, Changchun, China

**Keywords:** sleep, sleep disorder, mood disorder, neural mechanisms, therapy

Sleep and mental health are fundamentally and bidirectionally linked and coregulated. Patients with mood or mental disorders frequently exhibit sleep-wake dysregulation, while emotional dysfunction can contribute to the development and persistence of sleep disorders. Thus, elucidating the pathophysiological mechanisms underlying mood disorder-related sleep disturbances is paramount. This Research Topic comprises four articles that investigate the relationship between psychiatric conditions (including bipolar disorder, schizophrenia, and depression) and sleep disturbances, along with the potential impact of music therapy.

Diet- and metabolism-based interventions represent a promising, yet relatively underexplored frontier in the management of psychiatric syndromes characterized by concurrent sleep and mood disturbances. Researchers are increasingly investigating the application of the ketogenic diet to treat bipolar disorder and schizophrenia—conditions with high rates of comorbid sleep and circadian disruption. Preliminary evidence suggests that ketogenic interventions may modulate neurotransmitter dynamics, reduce oxidative stress, and stabilize the neural circuits involved in sleep regulation and emotional control. However, more comprehensive clinical studies are warranted to substantiate the therapeutic efficacy of this treatment, elucidate its underlying mechanisms, and evaluate its long-term safety and acceptability across different psychiatric populations.

Insomnia and depression are prevalent global disorders that often exist in a mutually exacerbating relationship. Contributing factors encompass genetic predisposition, hypothalamic-pituitary-adrenal (HPA) axis dysregulation, neuroinflammation, neuroendocrine perturbations, and alterations in the gut microbiota. Pathophysiologically, their co-occurrence signifies multisystem dysregulation spanning behavioral, neural, and molecular levels. These insights underscore the necessity of multifaceted, personalized preventive and therapeutic strategies that transcend isolated disease paradigms.

Sleep disturbances have deleterious effects that extend beyond mental health to encompass metabolic, cardiovascular, and immunological domains. Notably, sleep disturbance has been identified as a key mediator in the well-established association between depression and cardiovascular disease (CVD). Disrupted sleep can amplify physiological stress responses, contribute to hypertension, impair glycemic control, and promote low-grade systemic inflammation, each of which is a core pathophysiological mechanism in the development of CVD. Recognizing this mediatory role unveils novel opportunities for integrated preventive strategies targeting sleep to mitigate CVD risk in populations with depression.

While pharmacological and psychological interventions have traditionally dominated clinical management, music therapy is emerging as a promising, non-invasive, and patient-preferred adjunctive treatment for improving sleep disturbances. Music can reduce hyperarousal, modulate neuroendocrine and stress pathways (e.g., by decreasing cortisol levels and sympathetic activity), facilitate emotional processing, and promote positive mood states. Future research must address the heterogeneity of sleep and psychiatric disorders by tailoring music therapy interventions to the characteristics, symptom profiles, and cultural backgrounds of individual patients.

The relationship between mood and sleep disorders is more complex than mere comorbidity. It is rooted in the shared neural substrates that govern circadian rhythms, arousal, emotional processing, and metabolism. Progress demands a paradigm shift from treating discrete disorders to targeting their common underlying neural mechanisms. Combining cutting-edge neuroscience tools with innovative interventions such as metabolic therapies and neuromodulation, could break the cycle of debilitating symptoms and restore balance within the complex neural networks where mood and sleep converge. Advancing this field requires interdisciplinary collaboration to decode the brain’s mechanisms of sleep-emotion integration. Rigorous, longitudinal, multicenter investigations are essential to validate these innovative approaches, elucidate causal pathways, and develop personalized, multimodal care models that incorporate biological, psychological, social, and lifestyle factors.

